# Alcohol at the London Hospital

**Published:** 1886-10-16

**Authors:** 


### Hospital Administration.

Continued from page 6.—Communications for this column should be addressed, "Administrator," care of the Editor of The Hospital.

### ALCOHOL AT THE LONDON HOSPITAL, 1876 TO 1885.

**Reasons given.** In our last article on Alcohol, we stated that at the London Hospital, to which Mr. Sturge seemed to refer, the consumption of alcohol by the staff did not probably exceed the cost of a glass of bitter beer per head per diem. It will be seen by the figures in the following Table that the actual cost in 1885, including the money issued to certain servants in lieu of beer, amounts to rather less than thirty-six shillings per head per annum, or to just under three halfpence per diem. It may be asked, why do you select the London Hospital only? For the reasons that it is the largest and one of the very best managed of the Metropolitan hospitals, that it has been specially mentioned more than once in this alcohol discussion, and that the returns given below are so full and exhaustive as to possess special value and interest. The returns from other hospitals will be given subsequently.

**Differences in Systems of Diet.** The Table shows how great a change has taken place in the system of diets used at the hospitals during the last ten years. In 1876 every patient on ordinary diet probably received as a matter of course an allowance of beer or stout. Thus the amount spent upon malt liquors in 1876, when the staff and patients numbered together 7,066 persons, was £1,037, whereas only £685 was so spent in 1885, although the numbers had increased to 9,021. Again, in 1876 the sum spent upon milk was £1,847, whilst milk cost no less than £2,719 in 1885. This change in the incident of expenditure is largely, if not mainly, due to an alteration in the diet-table, and all who desire to arrive at the facts must keep this circumstance constantly in mind.

**Reduction in Expenditure on Alcohol.** It will be noticed that there has been an enormous reduction in the expenditure on alcohol during the last ten years. In 1876 the expenditure upon wine was £214, on spirits £625, and on malt liquors £1,110; total, £1,949, as against £118 on wine, £407 on spirits, and £869 on malt liquors; total, £1,394 in 1885. That is to say, whereas the cost of alcohol, including the staff and patients, was 3*s.* $1\frac{1}{10}$*d.* per head in 1885, it was no less than 5*s.* $6\frac{1}{4}$*d.* per head in the year 1876. It has been urged by some that the reduction here shown is not actual, but only apparent, because, whilst the quantity of alcoholic drinks consumed has undoubtedly decreased, considerable quantities of alcohol have been ordered for the patients in the form of spirits of wine. It may be well to state, therefore, that the amount expended upon spirits of wine in 1876 was £417, or 1*s.* $2\frac{1}{2}$*d.* per patient, whilst the expenditure in 1885 was £767, or 1*s.* $9\frac{1}{11}$*d.* That is to say, the whole cost of alcohol per head at the London Hospital fell from 6*s.* $8\frac{3}{4}$*d.* per head in 1876 to 4*s.* $10\frac{1}{2}$*d.* per head in 1885, a reduction of about thirty per cent.

### THE COST OF ALCOHOLIC STIMULANTS AT THE LONDON HOSPITAL.

| Year. | No. of Staff. | No. of Patients (under treatment). | Staff. Beer only. No Wine or Spirits allowed to Staff.* | Patients. Ordered as Medicine for Patients. Wine. | Spirits. | Beer. | Total. | Staff and Patients together. Wine. | Spirits. | Beer. | Total. |
|---|---|---|---|---|---|---|---|---|---|---|---|
| | | | £ s. d. | £ s. d. | £ s. d. | £ s. d. | £ s. d. | £ s. d. | £ s. d. | £ s. d. | £ s. d. |
| 1876 | 220† | 6846 | 1037 3 2 | 214 8 1 | 625 14 10 | 72 19 4 | 913 2 3 | 214 8 1 | 625 14 10 | 1110 2 6 | 1950 5 5 |
| 1877 | .. | 7438 | 930 11 6 | 128 6 11 | 334 5 4 | 125 3 6 | 587 15 9 | 128 6 11 | 334 5 4 | 1055 15 0 | 1518 7 3 |
| 1878 | .. | 7055 | 783 18 11 | 116 17 10 | 244 6 3 | 118 9 1 | 479 13 2 | 116 17 10 | 244 6 3 | 902 8 0 | 1263 12 1 |
| 1879 | 241† | 6361 | 701 2 0 | 140 2 1 | 238 13 6 | 162 13 2 | 541 8 9 | 140 2 1 | 238 13 6 | 863 15 2 | 1242 10 9 |
| 1880 | .. | 6844 | 670 3 9 | 130 10 4 | 374 12 9 | 243 2 4 | 748 5 5 | 130 10 4 | 374 12 9 | 913 6 1 | 1418 9 2 |
| 1881 | .. | 7394 | 677 15 3 | 157 18 4 | 306 7 10 | 211 6 0 | 675 12 2 | 157 18 4 | 306 7 10 | 889 1 3 | 1353 7 5 |
| 1882 | .. | 7798 | 687 11 10 | 106 0 8 | 302 0 3 | 125 7 4 | 533 8 3 | 106 0 8 | 302 0 3 | 812 19 2 | 1221 0 1 |
| 1883 | .. | 7976 | 711 13 7 | 136 8 8 | 409 12 7 | 150 9 1 | 696 10 4 | 136 8 8 | 409 12 7 | 862 2 8 | 1408 3 11 |
| 1884 | 290† | 8565 | 684 19 1 | 139 0 4 | 368 4 11 | 158 2 3 | 665 7 6 | 139 0 4 | 368 4 11 | 843 1 4 | 1450 6 7 |
| 1885 | 289† | 8732 | 685 0 1 | 118 2 9 | 407 1 10 | 184 3 1 | 709 7 8 | 118 2 9 | 407 1 10 | 869 3 2 | 1394 7 9 |

* Prior to 1885 no separate account was kept of Beer for staff and beer for patients, *consumed as diet.* Of that medicinally ordered for patients, as well as of wines and spirits so ordered, a separate account has always been kept. I have given the beer return for 1885 (for purposes of comparison) as for the previous years; but a dissected beer, wine, and spirit account for 1885 would stand as under—

| | | |
|---|---|---|
| 1885—Beer consumed by officers, nurses, and servants (male and female) .. .. | £389 9 4 | |
| Money issued to certain servants in lieu of beer .. .. .. .. | 130 11 4 | |
| | | £520 0 8 |
| Beer consumed by patients as diet .. .. .. .. .. | 164 19 5 | |
| Beer ordered for patients as medicine .. .. .. .. .. | 184 3 1 | |
| Wine ditto ditto .. .. .. .. .. | 118 2 9 | |
| Spirits ditto ditto .. .. .. .. .. | 407 1 10 | |
| | | £874 7 1 |
| Total as above .. .. .. .. | | £1394 7 9 |

† Prior to 1884 no collective return of the *numbers of the staff* was kept. I have, however, quoted the numbers as ascertained for special purposes in 1876 and 1879. The additional numbers of the years 1884-5 are mainly due to the increase of the nursing staff (which commenced about 1880) and to the appointment of ward maids. The medical and surgical visiting staff are of course not included in the numbers quoted.

(Signed) WM. J. NIXON, *House Governor.*

